# Alloying Bi-Doped Cs_2_Ag_1–*x*_Na_*x*_InCl_6_ Nanocrystals with K^+^ Cations Modulates Surface Ligands Density and Photoluminescence Efficiency

**DOI:** 10.1021/acs.nanolett.2c03112

**Published:** 2022-10-26

**Authors:** Zheming Liu, Juliette Zito, Michele Ghini, Luca Goldoni, Mirko Prato, Houman Bahmani Jalali, Ivan Infante, Luca De Trizio, Liberato Manna

**Affiliations:** ^†^Nanochemistry, ^§^Functional Nanosystems, ^∥^Materials Characterization, and ^⊥^Photonic Nanomaterials, Istituto Italiano di Tecnologia, Via Morego 30, 16163 Genova, Italy; ¶ Dipartimento di Chimica e Chimica Industriale, Università degli Studi di Genova, Via Dodecaneso 31, 16146 Genova, Italy

**Keywords:** double perovskites, doping, colloidal nanocrystals, trapped exciton, density functional theory calculations, ligand shell

## Abstract

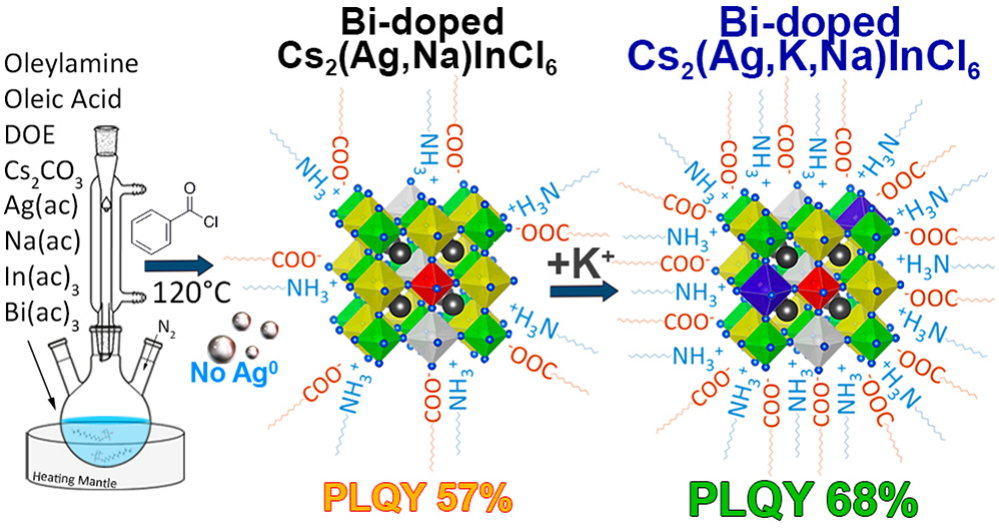

We show how, in the synthesis of yellow-emissive Bi-doped Cs_2_Ag_1–*x*_Na_*x*_InCl_6_ double perovskite nanocrystals (NCs), preventing the transient formation of Ag^0^ particles increases the photoluminescence quantum yield (PLQY) of the NCs from ∼30% to ∼60%. Calculations indicate that the presence of even a single Ag^0^ species on the surface of a NC introduces deep trap states. The PL efficiency of these NCs is further increased to ∼70% by partial replacement of Na^+^ with K^+^ ions, up to a 7% K content, due to a lattice expansion that promotes a more favorable ligands packing on the NC surface, hence better surface passivation. A further increase in K^+^ lowers the PLQY, due to both the activation of nonradiative quenching channels and a lower oscillator strength of the BiCl_6_→AgCl_6_ transition (through which PL emission occurs). The work indicates how a deeper understanding of parameters influencing carrier trapping/relaxation can boost the PLQY of double perovskites NCs.

The search for halide perovskite nanocrystal (NC) materials alternative to Pb-based compositions has led to the discovery of several potential candidates.^1−5^ Some of the most interesting compounds identified so far belong to the double-perovskite (DP) family and are characterized by a general A_2_B^+^B^3+^Cl_6_ stoichiometry.^2,3,6,7^ Examples are Cs_2_AgBiCl_6_, Cs_2_AgInCl_6_, Cs_2_NaInCl_6_, Cs_2_NaBiCl_6_, and Cs_2_AgSbCl_6_ NCs.^8−28^ These materials generally exhibit a weak photoluminescence (PL) due to the presence of an indirect bandgap or a direct bandgap with a parity forbidden band-edge transition.^3,6,7,21,29−32^ However, when appropriately doped/alloyed, they can become efficient emitters.^12,16−18,23,26−28,30,33−41^ For example, Mn^2+^- or Bi^3+^-doped Cs_2_AgInCl_6_ NCs and Mn^2+^-doped Cs_2_AgInCl_6_ NCs have an orange emission with a PL quantum yield (QY) up to ∼16%.^16,28^ If codoped with Ce^3+^ and Bi^3+^ cations, the PLQY of Cs_2_AgInCl_6_ NCs could be improved up to 26%, with the emission peaking at 580 nm.^42^ The introduction of Sb^3+^ dopants in Cs_2_NaInCl_6_ and Cs_2_KInCl_6_ NCs induces a broad blue-green emission centered at ∼450 nm with a PLQY up to ∼90%.^43,44^ The use of rare earth dopants in Cs_2_AgInCl_6_ or Cs_2_AgBiCl_6_ NCs confers them a near-infrared PL emission (∼1000 nm with Yb^3+^ and ∼1537 nm with Er^3+^).^17,18,36^

In this context, one of the most interesting systems is represented by Cs_2_NaInCl_6_, which can be doped with Bi^3+^ cations and alloyed with Ag^+^ ions to obtain Bi-doped Cs_2_Ag_1–*x*_Na_*x*_InCl_6_ materials featuring a broad and bright yellow emission with a PLQY as high as 86% in the bulk.^27,39,40^ Based on our previous density functional theory (DFT) calculations,^27^ the copresence of both Bi^3+^ and Ag^+^ cations is a prerequisite to achieve an efficient PL emission, which stems from a localized BiCl_6_→AgCl_6_ transition.^27,40^ As evidenced in another study, the PLQY of such NCs, synthesized with an optimal combination of surface ligands, is still much lower (37%) than that of the bulk counterpart.^40^ Such a difference has been attributed to the presence of deep trap states originating from unpassivated/undercoordinated surface Cl atoms.^40,43^ This explanation is supported by a recent work of Wang et al., who employed GeCl_4_ as the Cl precursor for the synthesis of Bi-doped Cs_2_Ag_1–*x*_Na_*x*_InCl_6_ NCs and reported a PLQY of ∼60% that was ascribed to the formation of NCs with Cl-rich surfaces, most likely with a low density of unpassivated surface Cl atoms.^44^ Recently, Levy et al. indicated that an additional cause of PL drop in these DP NCs can be the formation of Ag^0^ particles prior to the nucleation of the NCs.^45^ Specifically, they observed that in the synthesis of several Ag-based materials, namely Cs_2_AgMCl_6_ (M = In, Sb or Bi), the nucleation of the NCs is preceded by that of silver particles through the reduction of Ag^+^ cations by the alkylamines that are usually employed as surfactants. Likewise, we hypothesize that residual Ag^0^ species present on the surface of the Bi-doped Cs_2_Ag_1–*x*_Na_*x*_InCl_6_ NCs might affect their optical properties.

With the aim of further increasing the PLQY of Bi-doped Cs_2_Ag_1–*x*_Na_*x*_InCl_6_ NCs to approach bulk efficiencies, in this work we first sought to identify under which conditions Ag^0^ particles form prior to the nucleation of the NCs. Our results indicate that, under the typical reaction conditions (solubilization of metal acetates in oleic acid and oleylamine and then triggering the NCs nucleation by injecting benzoyl chloride, Bz-Cl^16,27,40,45^), Ag^0^ particles can form before the nucleation of the NCs takes place if the solubilization of precursors is performed at temperatures above 90 °C (Scheme 1).^45^ On the other hand, we discovered that, if the precursors are solubilized at 75 °C, the formation of Ag^0^ particles can be avoided. Interestingly, regardless of which synthesis route was followed, the DP NCs featured similar compositions, with no presence of metallic silver atoms, as assessed by X-ray photoelectron spectroscopy (XPS). Yet, the NCs synthesized by preventing the transient formation of Ag^0^ particles had a markedly higher PLQY (∼57 ± 5% versus 34 ± 3% of the NCs prepared with the transient formation of Ag^0^ particles). DFT calculations indicated that even tiny amounts of Ag^0^ present at the surface of NCs, down to 1 atom per NC, hence undetectable by techniques such as XPS, lead to efficient nonradiative trapping channels.

**Scheme 1:**
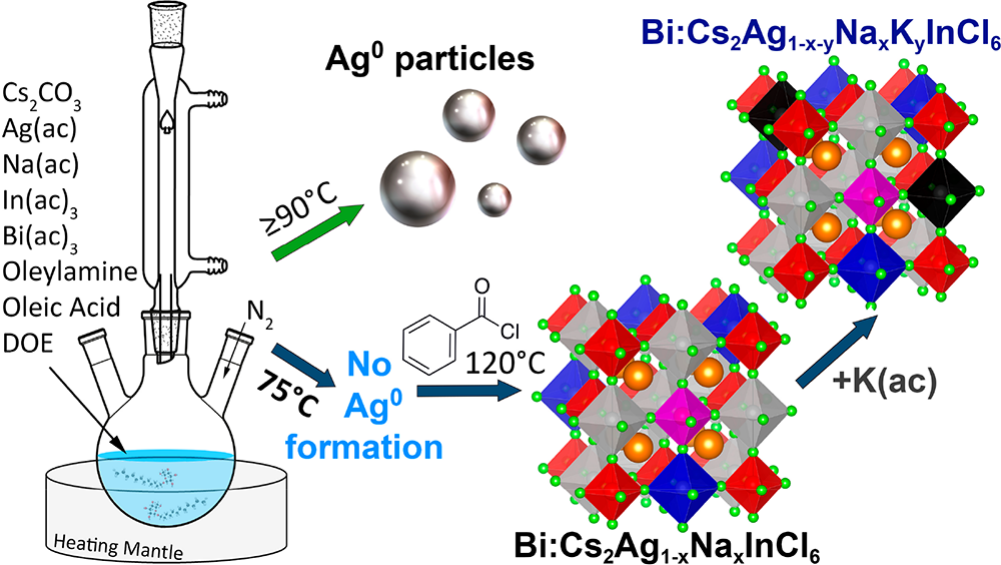
Reaction Scheme Developed for the Synthesis of Bi-doped Cs_2_Ag_1–*x*–*y*_Na_*x*_K_*y*_InCl_6_ NCs That Avoids the Prenucleation of Ag^0^ Particles

We then further attempted to mitigate the detrimental effects of undercoordinated Cl surface sites in Bi-doped Cs_2_Ag_1–*x*_Na_*x*_InCl_6_ NCs. One strategy we followed was to alloy Ag^+^ and Na^+^ with K^+^ cations in the B^+^ sites of the NCs, that is, to prepare Bi-doped Cs_2_Ag_1–*x*–*y*_Na_*x*_K_*y*_InCl_6_ NCs. The choice on potassium was inspired by several reports indicating that K^+^ cations can be used in the synthesis of halide perovskite NCs to achieve an efficient surface passivation, which, in turn, confers improved stability and higher PL efficiency.^46−48^ We observed that the incorporation of a small amount of K^+^ ions in the DP NCs, grown in the absence of Ag^0^ species, delivers Bi-doped Cs_1.92_Ag_0.20_Na_0.75_K_0.07_InCl_6.00_ NCs with a PLQY as high as 68%, a record value for such a NC system. However, a further increase in K^+^ content in the NCs resulted in a drop of the PLQY. Through nuclear magnetic resonance (NMR) analyses coupled with atomistic NC models, we could ascribe the improved PLQY at low K concentrations to an optimal ligand surface passivation, likely due to a more favorable interligand packing made possible by a slight increase in the lattice parameters induced by K^+^ ions (which are bigger than Na^+^ ions). Additionally, by DFT, we were able to ascribe the lower PLQY of the DP system with high K content to two possible causes: (i) a lower oscillator strength for the BiCl_6_→AgCl_6_ transition; (ii) local orthorhombic distortions occurring during the photoexcitation, which can activate efficient nonradiative quenching channels.

Our work not only allows one to synthesize colloidal DP NCs with a bright PL but also provides new insights on the passivation of trap states in metal halide NCs that are useful to maximize the emission efficiency of these systems.

We first investigated if and under what conditions Ag^0^ particles form during the synthesis of Bi-doped Cs_2_Ag_1–*x*_Na_*x*_InCl_6_ NCs. To do so, we employed the synthesis procedure previously reported by our group,^27,40^ and carefully varied the temperature at which the precursors are solubilized before the injection of Bz-Cl. We then examined the reaction mixture via optical and X-ray diffraction (XRD) analyses. We found that heating the reaction mixture at temperatures above 90 °C even for a short time (5 min) leads to the formation of Ag^0^ particles, as revealed by the appearance of a localized surface plasmon resonance absorption peak at 425 nm^45^ and further confirmed by XRD, which evidenced the presence of metallic Ag^0^ (Figure S1). Interestingly, even when Ag^0^ particles formed at the initial stage of the synthesis, no trace of metallic silver was found, neither by optical nor by XPS analyses of the final DP NCs (Figures S2 and S3). On the other hand, we observed that heating the reaction mixture at slightly lower temperatures, ∼75 °C, even for 10 min, leads to the solubilization of the precursors with no formation of metallic Ag^0^ (Figure S1). An additional rapid increase of the temperature up to 120 °C (at 45 °C/min), followed by the injection of Bz-Cl, promoted the nucleation of DP NCs without any intermediate formation of Ag^0^ particles (Figure S1). The Bi-doped Cs_2_Ag_0.15_Na_0.85_InCl_6_ NCs obtained in this way featured a bright yellow emission, with a PLQY as high as ∼57 ± 5% (Figure 2e,g), a value that is much higher than that of the NCs made in the presence of Ag^0^ (i.e., 34 ± 3%, Figure S3).^40^ Such a procedure was also observed to yield a better control over the size distribution of the DP NCs, with a mean size of ∼10.5 ± 1.1 nm (∼10% size dispersion) while the previously reported procedure delivered ∼16.5 ± 2.5 nm particles (∼15% size dispersion) (Figures S3 and S4).

These results indicate that the formation of Ag^0^ particles prior to the nucleation and growth of Bi-doped Cs_2_Ag_1–*x*_Na_*x*_InCl_6_ NCs has a negative impact on their optical properties. While XRD and XPS analyses did not reveal any difference between the DP NCs prepared using the two synthetic procedures (Figure S2), we hypothesize that residual Ag^0^ atoms present after the dissolution of preformed Ag^0^ particles, even in amounts below the detection limits of XPS,^49^ can still affect the optical properties of the final NCs. To support this hypothesis, we performed DFT calculations on a ∼3.5 nm Cs_2_Ag_0.60_Na_0.40_InCl_6_ NC model^40^ to the surface of which we added one Ag atom, while keeping the system charge neutral. This addition results in the emergence of a deep trap state spatially localized at the added Ag, thus formally generating a metallic Ag^0^ at the surface (Figure 1). This suggests that, even in trace amounts (as low as 1 atom per NC), the presence of residual amounts of metallic Ag^0^ at the NC surface promotes the formation of efficient nonradiative trapping channels.

**Figure 1:**
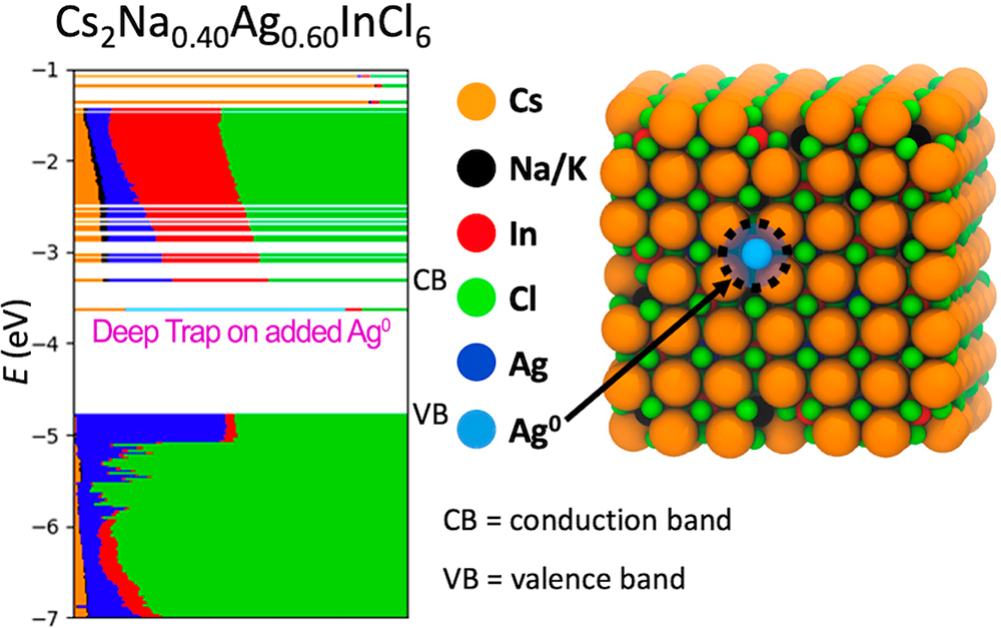
On the left, the electronic structure of the reduced Cs_2_Ag_0.60_Na_0.40_InCl_6_ NC model computed at the DFT/PBE level of theory. The outermost unpaired electron is localized at the added Ag atom, which formally becomes a metallic Ag atom creating a deep trap state. On the right, the relaxed structure of the DP NC model with the position of the added Ag circled in black.

In the second part of this work, we adopted the optimized synthesis protocol, which avoids the Ag^0^ formation, and we sought to further improve the PLQY of Bi-doped Cs_2_Ag_1–*x*_Na_*x*_InCl_6_ NCs by alloying with K^+^ cations. In detail, we fixed the amount of Bi and Ag, by keeping Bi/In = 0.5% and Ag/In = 0.15/1 (as they were observed to optimize the PL emission, see Figure S5) and we systematically varied the K/Na/In precursors ratios from 0/0.85/1 to 0.85/0/1 (Table 1). The NCs obtained with our optimized procedure have a cubic shape and a mean size of 10–12 nm (Figure 2a,b and Figure S4). The compositional analysis, performed by coupling XPS and inductively coupled plasma (ICP) optical emission spectrometry (OES) (the latter employed to measure the %Bi doping) indicated the formation of Bi-doped Cs_2_Ag_1–*x*–*y*_Na_*x*_K_*y*_Cl_6_ NCs, with variable amounts of K cations (Table 1).

**Table 1:** Composition of Bi-Doped Cs_2_Ag_1–*x*–*y*_Na_*x*_K_*y*_InCl_6_ NCs Measured via XPS and ICP-OES Analyses

Na/K/In precursor ratio	Stoichiometry	Bi/In (%)	Sample Name
0.85/0/1	Cs_1.76_Ag_0.21_Na_0.74_InCl_5.78_	0.47	0%K
0.8/0.05/1	Cs_1.92_Ag_0.20_Na_0.75_K_0.07_InCl_6.00_	0.47	7%K
0.75/0.1/1	Cs_2.03_Ag_0.13_Na_0.76_K_0.14_InCl_5.84_	0.62	14%K
0.65/0.2/1	Cs_1.90_Ag_0.17_Na_0.58_K_0.22_InCl_5.95_	0.57	22%K
0/0.85/1	Cs_1.63_Ag_0.12_K_0.89_InCl_5.37_	0.54	89%K

**Figure 2:**
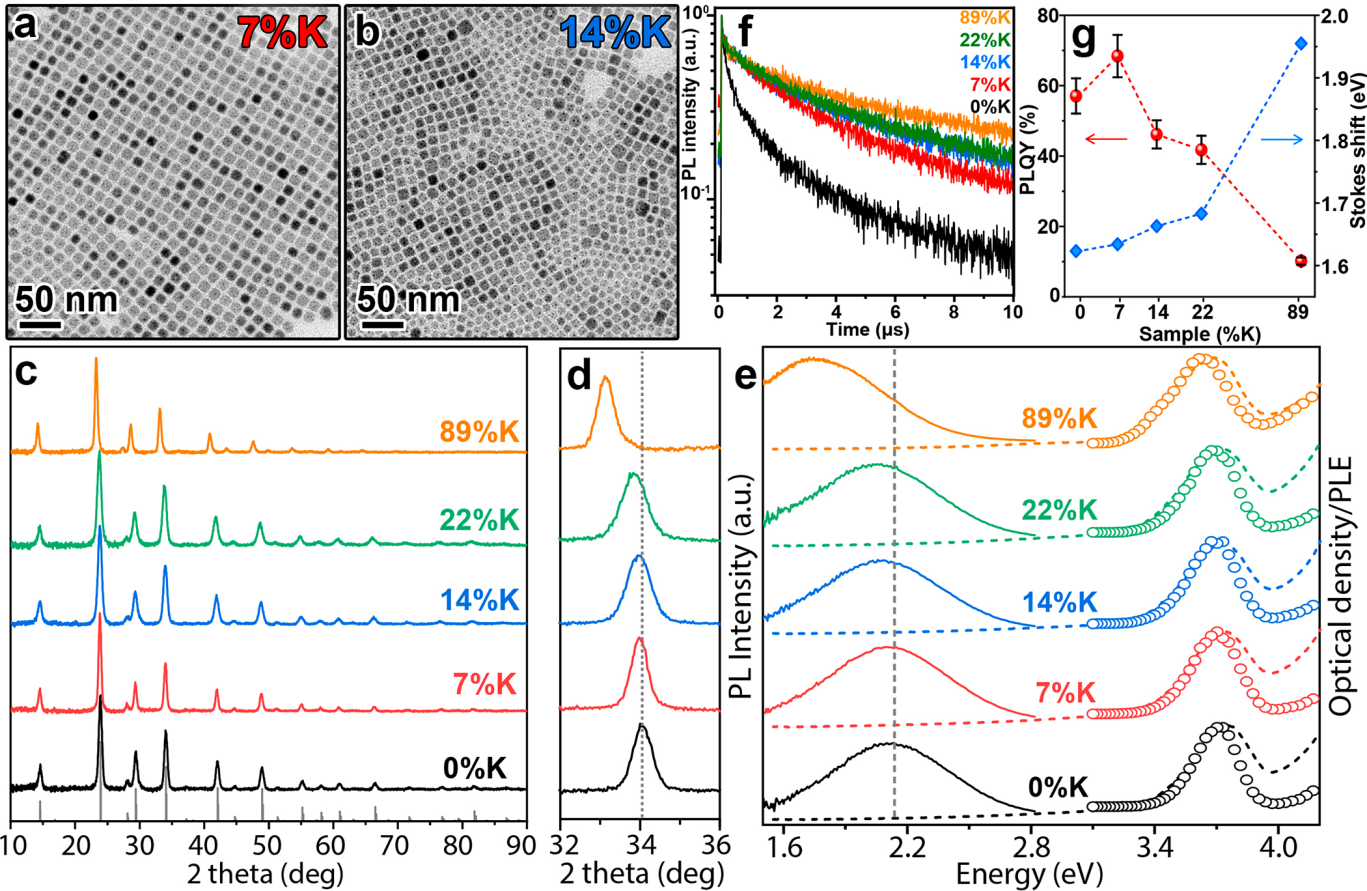
TEM images of (a) 7%K and (b) 14%K NC samples. (c) XRD patterns (with the magnification of the 32°–36° range (d)), (e) absorption, PL and PLE spectra, (f) normalized time-resolved PL decay traces, (g) PLQY and Stokes shift values of Bi-doped Cs_2_Ag_1–*x*–*y*_Na_*x*_K_*y*_InCl_6_ NCs (samples names are reported in Table 1).

The XRD patterns of these samples could be all indexed with the expected cubic Cs_2_NaInCl_6_ DP structure (ICSD number 132718), with no presence of secondary phases (Figure 2c). Interestingly, a slight shift toward lower 2θ angles was observed along with the increase in K content in the DP NCs (Figure 2d). This is consistent with the larger ionic radius of K^+^ (138 pm) compared to that of Na^+^ (102 pm) in octahedral coordination.^50^ These structural characterizations indicate the successful formation of alloyed Bi-doped Cs_2_Ag_1–*x*–*y*_Na_*x*_K_*y*_InCl_6_ NC structures.

The effects of inserting K^+^ ions in the DP NCs were investigated via optical analyses (Figure 2e–g). All the samples were characterized by an absorption peak at ∼3.72 eV, in agreement with previous reports,^27,40^ and a broad PL emission whose position was observed to shift to lower energies (resulting in a higher Stokes shift) when increasing the K content (Figure 2e). In detail, the PL peak position of the 0%K NC sample was 2.11 eV and shifted to 2.04 eV upon incorporation of 22% of K and to 1.73 eV in the 89%K sample (the one prepared in the absence of Na). The time-resolved PL decay traces indicated that the PL lifetimes of the samples increased together with the amount of K incorporated, from 2.4 μs (0%K) to 4.2 μs (89%K) (Figure 2f and Table S1). Interestingly, the PLQY of the samples increased up to ∼68% ± 6% for the 7%K sample and then systematically decreased when the K content (Figure 2g) was increased. Moreover, the NC samples (namely 0%K and 7%K) were observed to be stable for up to 2 weeks when exposed to air (Figure S6).

In order to rationalize the optical features and to explain the role played by K^+^ in the DP NCs, we performed DFT calculations. These were done through a side-by-side atomistic investigation of Bi-doped Cs_2_Ag_1–*x*_Na_*x*_InCl_6_ and Cs_2_Ag_1–*y*_K_*y*_InCl_6_ model. We started our comparison by running calculations on bulk Cs_2_NaInCl_6_ and Cs_2_KInCl_6_ cubic unit cells in which either 1 Na^+^ or 1 K^+^ ion are replaced by 1 Ag^+^ ion and 1 In^3+^ ion is replaced by 1 Bi^3+^ ion (Figure 3a). Although these models overestimate the Bi^3+^ concentration, their limited size enabled us to include spin–orbit coupling in the calculations. As highlighted in Figure 3a,^27^ the addition of spin–orbit coupling resulted in an important stabilization of the antibonding Bi(6p)–Cl(3p) orbitals, which consequently dominate the conduction band minimum at the M point. On the other hand, the valence band maximum is formed by antibonding Ag(5d)–Cl(3p) orbitals and lies at the Γ point.^27^ These new calculations support the trapped exciton picture proposed for Bi-doped Cs_2_Ag_1–*x*_Na_*x*_InCl_6_ systems, with the photogenerated carriers being localized at the BiCl_6_ and AgCl_6_ centers (Figure 3a, right panel).^27^ Interestingly, the band structures of Cs_2_NaInCl_6_ and Cs_2_KInCl_6_ codoped with Ag^+^ and Bi^3+^ ions look very similar to each other, with only a negligible contribution of Na^+^ and K^+^ ions to the band edge states.

**Figure 3:**
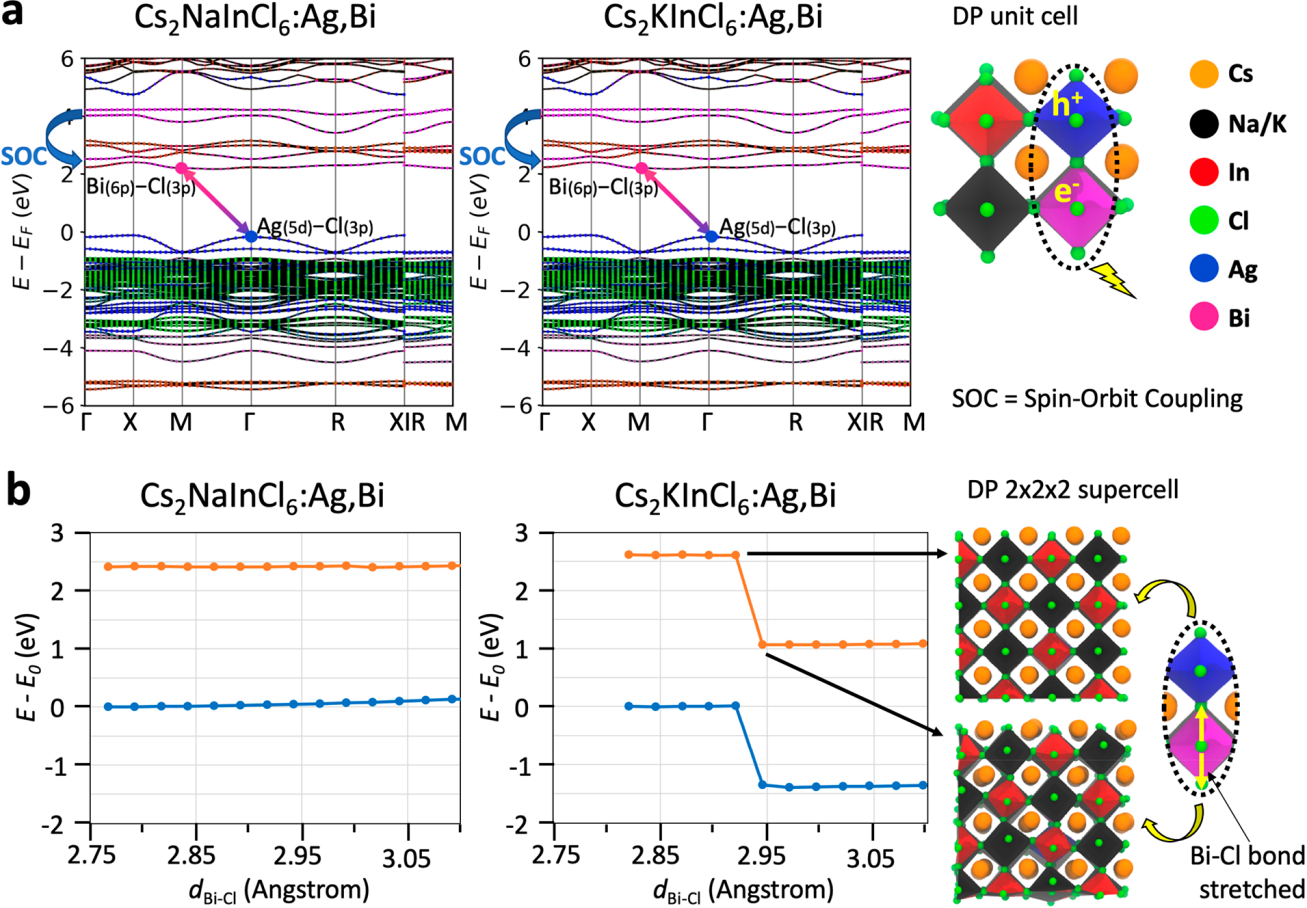
(a) Band structure of the Cs_2_NaInCl_6_ and Cs_2_KInCl_6_ bulk systems doped with 1 Ag^+^ and 1 Bi^3+^ ions, computed at the DFT/PBE level of theory on the 1 × 1 × 1 unit cell. Each band is decomposed according to each atom type. (b) Trend of the ground state and triplet state (mimicking the first excited state) energies by stretching the Bi–Cl bond computed for both Cs_2_NaInCl_6_ and Cs_2_KInCl_6_ bulk systems (2 × 2 × 2 super cells) and doped with 1 Ag^+^ and 1 Bi^3+^ ions. The ground state relaxed structures of both systems are taken as the reference 0 energy values. For the K-based system, we observe a distortion from the cubic to the orthorhombic structure when the bond is stretched above a threshold length of 2.92 Å.

To account for the influence of the Na^+^ and K^+^ ions on the radiative recombination pathways of our systems, we then included 1 Ag^+^ ion and 1 Bi^3+^ ion in more realistic DP matrices consisting of 2 × 2 × 2 replications of the bulk Cs_2_NaInCl_6_ and Cs_2_KInCl_6_ unit cells, respectively. Here, we first computed the oscillator strength of the lowest BiCl_6_→AgCl_6_ electronic transition and found a lower value in the K-based system (*f* = 1.58) than in its Na-based counterpart (*f* = 2.11), in line with the observed increase in PL lifetime (Figure 2f) and the drop in PLQY (Figure 2g) observed at high concentrations of K^+^. For both systems, we also probed the extent of the Stokes shift by computing the ground state (singlet) and lowest excited state (triplet) potential energy surfaces (Figure 3b). We systematically elongated the Bi–Cl bond along the Bi–Cl–Ag axis to qualitatively follow the partial breaking of this bond upon photoexcitation, i.e., by occupying one of the 3-fold-degenerate Bi(6p)–Cl(3p) antibonding molecular orbitals. Irrespective of the stretching distance, the computed Stokes shift of the Cs_2_NaInCl_6_:Ag,Bi system was slightly lower than that of Cs_2_KInCl_6_:Ag,Bi (ca. −2.42 and −2.62 eV, respectively, Figure 3b), in agreement with the experimental trend (Figure 2e,g). Nonetheless, our calculations on the Cs_2_KInCl_6_:Ag,Bi system highlight a sudden stabilization in energy when the Bi–Cl bond length is stretched from 2.920 to 2.945 Å. A closer look at the supercell structures suggests a phase-transformation of the K-based DP matrix from cubic to orthorhombic (Figure 3b). Although this phase-transformation likely occurs only in the photoexcited state, and thus it is short-lived and challenging to be detected experimentally, we can expect that a high concentration of K^+^ ions around AgCl_6_–BiCl_6_ emission centers might promote local orthorhombic distortions upon photoexcitation, which is a prelude for an efficient nonradiative quenching mechanism due to a large energy (and structural) reorganization in the excited state. This effect is likely also responsible for the larger Stokes shift observed for K-based DP systems compared to the pure Na-based one.

We then investigated the reason behind the increase of PLQY at low levels of K^+^ incorporation in the DP NCs. Considering the low amount of K in the lattice, we did not expect the structural reorganizations in the excited state to play any role in the PL efficiency. On the other hand, we hypothesized that surface defects might differ when K^+^ is incorporated. To verify this, we monitored the variation in the electronic structure of ∼3.5 nm Cs_2_Ag_0.60_Na_0.40_InCl_6_ and Cs_2_Ag_0.60_K_0.40_InCl_6_ NC models upon systematic displacement of surface CsCl ion pairs to mimic the displacement of ionic ligand pairs (e.g., Cs-oleate) from the NC surface.^40^ This resulted in the appearance of deep Cl surface trap states above the valence band in both Na-based and K-based systems (Figure S7). Since we did not observe a notable difference between the energetic positions of these traps in the two systems, we could expect that their different PLQYs stem from the concentration of surface traps rather than whether traps are deeper or shallower.

The concentration of surface traps can be correlated to the extent of surface passivation. To this aim, we estimated the extent of surface passivation in 0%K and 7%K NC systems by taking into account both NMR and XPS analyses (Table 1). To evaluate the surface ligands coverage, we performed quantitative ^1^H NMR analyses on solutions obtained by dissolving the NC samples (0%K and 7%K) in dimethyl sulfoxide-*d*_6_ (Figures S8–10). This solvent dismantled the NCs and freed the ligands bound to their surface. Our results indicated that the ligands density of the 7%K NC sample (2.3 ligands/nm^2^) was much higher than that of the 0%K sample (1.3 ligands/nm^2^) (see SI for details on the calculations). Considering both size and composition measured for these two NC samples, such ligands densities correspond to 35% and 22% of surface A(Cs+Oleylammonium) and X(Cl+Oleate) sites occupied, respectively, in the 0%K system and to 88% and 57% for the 7%K NC sample (see the SI for details). Such improved surface passivation could be ascribed to the increase in lattice parameters induced by the incorporation of a small amount of K ions inside Bi-doped Cs_2_Ag_1–*x*_Na_*x*_InCl_6_ NCs (from *a* = 10.47 Å for 0%K to *a* = 10.53 Å for 7%K, Figure 2c,d), which is believed to promote a more favorable ligands packing that cannot be reached for smaller lattice parameters (that is, in the absence of K).

In conclusion, in this work, we have found that avoiding the nucleation of Ag^0^ particles during the synthesis of Bi-doped Cs_2_Ag_1–*x*_Na_*x*_InCl_6_ nanocrystals improves significantly their PLQY, as any residual Ag^0^ atoms on their surface can act as efficient nonradiative traps. We also identified the partial replacement of Na^+^ with K^+^ ions, up to 7%, as an efficient tool to boost the PLQY (reaching values close to 70%). This was rationalized as due to an expansion in the lattice parameters that enables a higher surface ligands density, that is a better passivation of surface sites. Further increases in the K^+^ content were found to be detrimental for the PLQY, as they decreased the oscillator strength for the optically active transition of the self-localized carriers and additionally activated nonradiative decay channels. The findings of the present work finally narrow the gap between bulk and nanosized Bi-doped Cs_2_Ag_1–*x*_Na_*x*_InCl_6_ in terms of optical performance and are likely to be extendable to other systems in addition to the double perovskites studied here: first, prenucleation of metallic species needs to be avoided as much as possible; second, introducing small amounts of cation substituents can be an efficient tool to better accommodate surface ligands, hence to passivate a higher number of surface states.

## Supporting Information Available

Experimental details, XRD and absorption curves, XPS spectra, TEM pictures, PLQY values, electronic structure calculations, NMR spectra, surface ligands density and sites occupancy (PDF)
