# Supplementary material for: Alloying Bi-Doped Cs2Ag1–xNaxInCl6 Nanocrystals with K+ Cations Modulates Surface Ligands Density and Photoluminescence Efficiency
Source: Nano Lett. 2022 Oct 26;22(21):8567–73. doi: 10.1021/acs.nanolett.2c03112 (PMC9650775; doi:10.1021/acs.nanolett.2c03112)
Supplement: Supplementary file 1 — nl2c03112_si_001.pdf [file nl2c03112_si_001.pdf]

SUPPORTING INFORMATION FOR:

Alloying Bi-doped $Cs_2Ag_{1-x}Na_xInCl_6$ Nanocrystals with K^+ Cations Modulates Surface Ligands Density and Photoluminescence Efficiency

Zheming Liu[‡a,b], Juliette Zito[‡a,b], Michele Ghini[c], Luca Goldoni[a], Mirko Prato[d], Houman Bahmani Jalali[a,e], Ivan Infante[a*], Luca De Trizio[a*], Liberato Manna[a*]

[a]Nanochemistry, [c]Functional Nanosystems, [d]Materials Characterization and [e]Photonic Nanomaterials, Istituto Italiano di Tecnologia, Via Morego 30, 16163 Genova, Italy

[b]Dipartimento di Chimica e Chimica Industriale, Università degli Studi di Genova, Via Dodecaneso 31, 16146 Genova, Italy

EXPERIMENTAL SECTION

Chemicals. Cesium carbonate (Cs_2CO_3, 99%), silver acetate (Ag(ac) 99.99%), sodium acetate (Na(ac), 99%), potassium acetate (K(ac), 99%), indium(III) acetate($In(ac)_3$, 99.99%), bismuth acetate ($Bi(ac)_3$, 99.99%), benzoyl chloride (Bz-Cl, 98%), dioctyl ether (DOE, 99%), oleic acid (OA, 90%), oleylamine (OLAM, 98%), toluene-d_8(anhydrous, 99.6%), dimethyl sulfoxide-d_6(DMSO-d_6, 99.9%), toluene (anhydrous), ethanol (anhydrous), hexane (anhydrous) and ethyl acetate (99.8%) were purchased from Sigma-Aldrich. All chemicals were used without any further purification.

Synthesis of $Cs_2Ag_{1-x-y}Na_xK_yInCl_6$ NCs. In a typical synthesis, Cs_2CO_3 (0.25 mmol), Ag(ac) ((1-x-y)*0.25 mmol), Na(ac) (x*0.25 mmol), K (ac) (y*0.25 mmol), $In(ac)_3$ (0.25mmol), $Bi(ac)_3$ (1.25 µmol, 0.5% with respect to $In(ac)_3$), degassed OLAM (0.5mL), degassed OA (1.2 mL) and DOE (4 mL) were mixed in a three neck flask under inert atmosphere (i.e. N_2) . The mixture was heated up to 75°C under vigorous stirring for 10 min to dissolve the metal cation precursors. Then, the system was quickly heated up to 120°C (at a rate of 45°C/min) and a solution of 220 µL Bz-Cl in 0.5 mL of degassed DOE was swiftly injected into the flask. Right after the injection, the reaction was cooled down by an ice-water bath. The crude reaction solution was centrifuged at 4000 rpm for 5 min and the supernatant was discarded. The precipitate was dispersed in 4 ml of hexane and then further cleaned by adding extra 8mL of ethyl acetate and the centrifuging at 4000rpm for 5min. The precipitated NCs were redispersed in 4ml of hexane and was stored in a vial for further characterizations. All the washing procedures were carried out under inert atmosphere. The synthesis procedure employed to monitor the formation of Ag^0 species prior the NCs formation was identical to the one just described, except that no K precursor was employed and the solubilization of the precursors (before the injection of Bz-Cl) was performed at 90°C.

X-ray Diffraction (XRD) Characterization. The XRD analysis was performed on PANanalytical Empyrean X-ray diffractometer equipped with a 1.8 kW Cu Kα ceramic X-ray tube and a PIXcel3D 2x2 area detector, operating at 45 kV and 40 mA. Specimens for the XRD measurements were prepared by dropping a concentrated NCs solution onto a silicon zero-diffraction single crystal

substrate. The diffraction patterns were measured under ambient conditions using a parallel beam geometry and the symmetric reflection mode in the range of 10°-90° 2 theta. XRD data analysis was performed using the HighScore 4.1 software from PANalytical.

Transmission Electron Microscopy (TEM) analysis. The TEM measurements were carried out on a JEOL JEM-1400Plus microscope with a thermionic gun (W filament) operated at an acceleration voltage of 120 kV. The specimens were prepared by dropping dilute NCs solutions onto 200 mesh carbon-coated copper grids for TEM.

Inductively Coupled Plasma (ICP-OES) Elemental Analysis. ICP elemental analysis, carried out via inductively coupled plasma optical emission spectroscopy (ICP-OES) with an iCAP 6300 DUO ICP-OES spectrometer (ThermoScientific) was used to quantify the Bi to In ratio. The samples were dissolved in 2.5 ml of aqua regia (HCl/HNO_3=3/1(v/v)) overnight and then were diluted by adding Milli-Q water to 25ml of solution. All chemical analyses performed by ICP-OES were affected by a systematic error of about 5%.

X-ray Photoelectron Spectroscopy (XPS) Characterization. XPS analysis was carried out on a Kratos Axis Ultra^{DLD} spectrometer using a monochromatic Al Kα source (20 mA, 15 kV). Survey scan analyses were carried out with an analysis area of 300 x 700 microns and a pass energy of 160 eV, whereas high resolution analyses were conducted with a pass energy of 10 eV. Specimens for XPS measurements were prepared by dropping a concentrated NC solution onto a freshly cleaved highly oriented pyrolytic graphite substrate (HOPG, ZYB). Spectra were analyzed using the CasaXPS software (version 2.3.24).[1]

Optical measurements. The UV−visible absorption spectra were recorded on a Varian Cary 300 UV−vis absorption spectrophotometer. PL, PLE and PLQY measurements were collected on an Edinburgh FLS920 spectrometer equipped with a continuous Xenon lamp (Xe900) and an integrating sphere. PLE and PL spectra were corrected by taking into account the lamp and setup response. For absolute PLQY measurements we used a correction file specifically calibrated for the integrating sphere, grating and detector employed. The samples were diluted to reach an optical density of 0.1-0.15 at the wavelength of excitation (335 nm). The time-resolved PL measurements were carried out on a Edinburgh FLS900 fluorescence spectrometer equipped with a time-correlated single photon counting unit coupled with a Edinburgh Instruments EPL-375 pulsed laser diode (λ_{ex} = 371.8 nm, pulse width = 68.8 ps). The lifetime values were calculated by Flouracle software.

Computational Methodology. Band structure calculations on Bi-doped $Cs_2Ag_xNa(K)_{1-x}InCl_6$ were performed on a 1x1x1 unit cell, using the VASP 5.2 package.[2-4] The exchange–correlation potential was approximated by the PBE exchange–correlation functional,[5] with the further inclusion of the spin–orbit coupling term within the noncollinear approximation. We used a *k* mesh grid of 4 × 4 × 4 for the Brillouin zone integration. The atomic positions were relaxed until the forces were smaller than 0.001 hartree/angstrom. We used a kinetic energy cutoff of 400 eV. Transition dipole moments for the lowest energy transition, on the other hand, were evaluated using the Fermi's golden rule and were performed on 2x2x2 supercells at the gamma point. Both atomic positions and cell parameters were relaxed at the DFT level using the PBE exchange−correlation functional and a double-ζ basis set plus polarization functions (DZVP) on all atoms,[6] as implemented in the CP2K 8.1 package.[7] Scalar relativistic effects were incorporated as effective core potentials.[6] The role of surface defects was probed by modeling explicit cubic $Cs_2Ag_xNa(K)_{1-x}InCl_6$ NCs of about 3.0 nm. NCs models were prepared by cutting the corresponding cubic bulk structures along the (100) facets, leaving Cs and Cl on the surface. As-cut, these nanostructures present a stoichiometry of $Cs_{343}Ag_xNa_{108-x}In_{108}Cl_{756}$, corresponding to an excess of positive charge when each ion is considered in its more stable thermodynamic electronic configuration (i.e. Cs^+, Na^+ and Ag^+, In^{3+} and Cl^-). This excess was compensated by removing 19 Cs ions from the surface, leading to a charge balanced $Cs_{324}Ag_xNa(K)_{108-x}In_{108}Cl_{756}$ stoichiometry. Structural relaxation and analysis of the electronic structure was carried out at the DFT/PBE/DZVP level of theory with CP2k.

Nuclear magnetic resonance (NMR) measurements. All 2D NMR spectra were acquired on a Bruker AvanceIII 600 MHz spectrometer, supplied with a cryoprobe whereas ^{1}H NMR spectra were collected on a Bruker AvanceIII 400 MHz spectrometer equipped with a triple resonance broadband NMR probe (iProbe TBO). All the NMR experiments were run at 298K. The matching and tuning, and the homogeneity was improved automatically, and the 90° pulse for ^{1}H was optimized by an automatic pulse calculation routine.[8] The ^{1}H NMR spectra in toluene-d$_8$, Figure S8 and in DMSO-d$_6$, Figure S9, were acquired by accumulating 64 transients without dummy scans, at a fixed receiver gain (64), by using an inter-pulses delay of 30 s and 65536 digit points, over a spectral width of 20.49 ppm, with the transmitter frequency offset positioned at 6.18 ppm. The ^{1}H-^{13}C Heteronuclear Single Quantum

Coherence (HSQC) experiment (multiplicity edited, "hsqcedetgpsp.3" pulse sequence of the Bruker library), Figure S10 i) was acquired with 8 FIDs, 2024 digit points, 518 increments and $^{1}J_{CH}$ = 145 Hz, over a spectral width of 13.02 ppm for ^{1}H and 165.0 ppm for ^{13}C, with the offset set at 6.40 and 75.0 ppm respectively. In the ^{1}H-^{13}C Heteronuclear Multiple Bond Correlation (HMBC) experiment ("hmbcgpndqf" pulse sequence of the Bruker library), Figure S10 ii), 256 FIDs, 4096 digit points and 128 increments were gathered with $^{1}J_{CH}$ long range =10 Hz, over a spectral width of 15.15 ppm and 220.0 ppm for ^{1}H and ^{13}C respectively, with the offset centered at 7 and 100 ppm. An exponential apodization function equivalent to 0.3 Hz was applied to the ^{1}H NMR FIDs (Free Induction Decay), prior to the Fourier transform. All the spectra were manually phased and the baseline was automatically corrected. The concentrations of oleylamine (OLAM) and oleic acid (OA) were measured by comparing the integrated peak intensity of diagnostic signals reported in Figure S8, normalized to the number of ^{1}H resonances generating the signal (i.e. 2H) to the signal (normalized to 2H, too) of a 10 mM solution in DMSO-d_6 of maleic acid (TraceCERT, Sigma-Aldrich 99.99%) for *q*NMR by means of the PULCON (PUlse Length Based Concentration Determination) external standard method.[9]

Bi-doped $Cs_2Ag_{1-x-y}Na_xK_yInCl_6$ NCs analyzed via NMR were washed through the following steps: (1) the crude reaction solution (6 ml) was centrifuged at 6000 rpm for 5 min. (2) The precipitate was dispersed in 2 ml of anhydrous toluene followed by vigorous shaking to get a turbid brown suspension; (3) The turbid suspension was left to sediment overnight and the transparent supernatant was then collected; (4) 2ml of ethyl acetate were added to 1ml of supernatant and then the mixture was centrifuged at 5000 rpm for 5 min; (5) the precipitate was dispersed in 1 ml of anhydrous toluene again and precipitated via the addition of 2ml of ethyl acetate and centrifugation at 5000rpm for 5min; (6) the precipitate was collected and redispered in 1 ml of toluene-d_8.

^{1}H NMR spectra of the NC samples were performed in toluene-d_6 before their dissolution in DMSO-d_6 to evaluate the quality of the samples in order to exclude impurities deriving from the synthesis e.g. solvent residue.

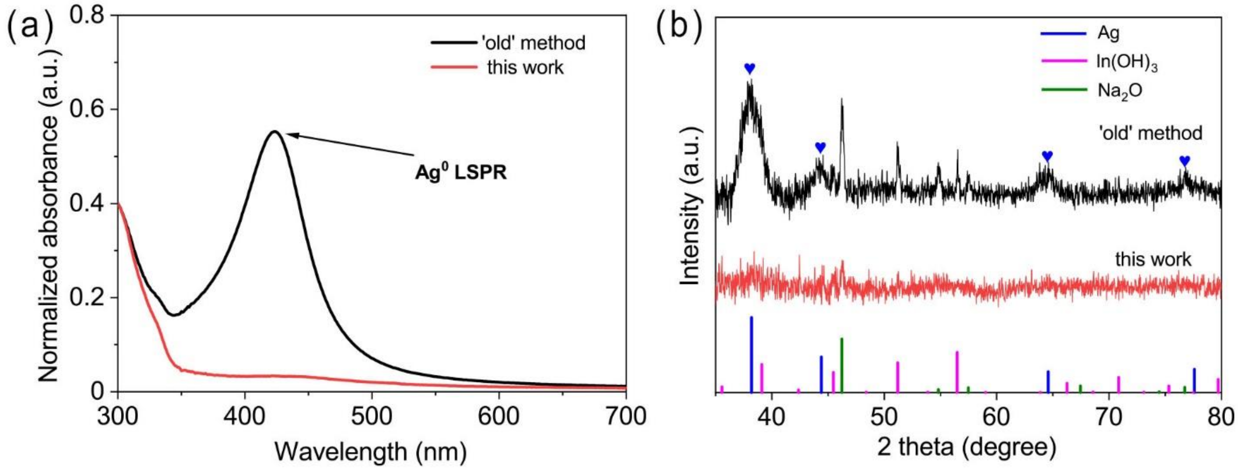

Figure S1. (a) Optical absorption spectra and (b) XRD patterns of precursor mixtures employed for the synthesis of Bi-doped $Cs_2Ag_{0.15}Na_{0.85}InCl_6$ nanocrystals with either our previously reported method (in which the precursors are solubilized at 90°C for 5min),[10] or the strategy devised in the current work (where the precursors are dissolved at 75°C for 10min and then the temperature was quickly raised up to 120°C). In both cases, after dissolving the precursors instead of injecting the benzoyl chloride, the reaction mixture was cooled down to room temperature with an ice-water bath. The absorption spectra were measured by diluting the precursors mixture in toluene. The localized surface plasmon resonance (LSPR) absorption of Ag^0 particles, peaked at 425nm, is clearly visible in the absorption curve (black line, panel a) of the precursors mixture prepared following our previously reported method. To prepare the XRD specimens, 3ml of the reaction mixture was mixed with 21ml of anhydrous ethanol and centrifuged at 10000rpm for 10min at 10°C. The supernatant was discarded and the precipitate was analyzed. The bulk reflections in panel (b) correspond to Ag (ICSD 96-901-2432), Na_2O (ICSD 96-101-0877) and $In(OH)_3$ (ICSD 98-003-5637).

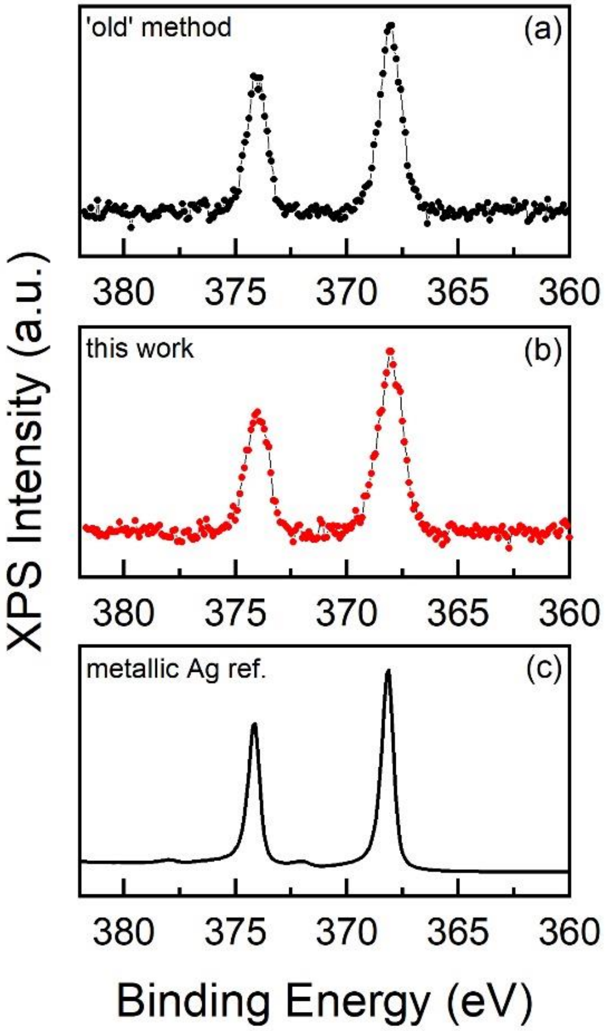

Figure S2. XPS Ag 3d spectra collected on Bi-doped $Cs_2Ag_{0.15}Na_{0.85}InCl_6$ nanocrystals prepared with either (a) our previously reported method (in which the precursors are solubilized at 90°C for 5min),[10] or (b) the strategy devised in the current work (where the precursors are dissolved at 75°C for 10min and then the temperature was quickly raised up to 120°C). In both cases, the main Ag peak (Ag $3d_{5/2}$) is centered at (368.0±0.2) eV, in line with reports on Ag(I) compounds.[11] Panel c reports, for comparison, the Ag 3d spectrum collected on a freshly cleaned (by Ar^+ ion sputtering) Ag foil. The spectrum shows the presence of two intense, narrow, and slightly asymmetric peaks, separated by 6 eV, typical of Ag^0, together with low-intensity loss features at the higher binding energy side of each spin-orbit component. The position of the Ag $3d_{5/2}$ is (368.1±0.2) eV, in agreement with literature reports on Ag^0.[12] Given the observed positions, it is hardly possible to determine by XPS if Ag^0 is present in our DP NCs. However, the similarity between the spectra of panels (a) and (b) suggests that, within the detection limits of XPS, the oxidation state of Ag in the two samples is the same (and it is Ag^+, given peak width, symmetry and the absence of high-binding-energy loss features).

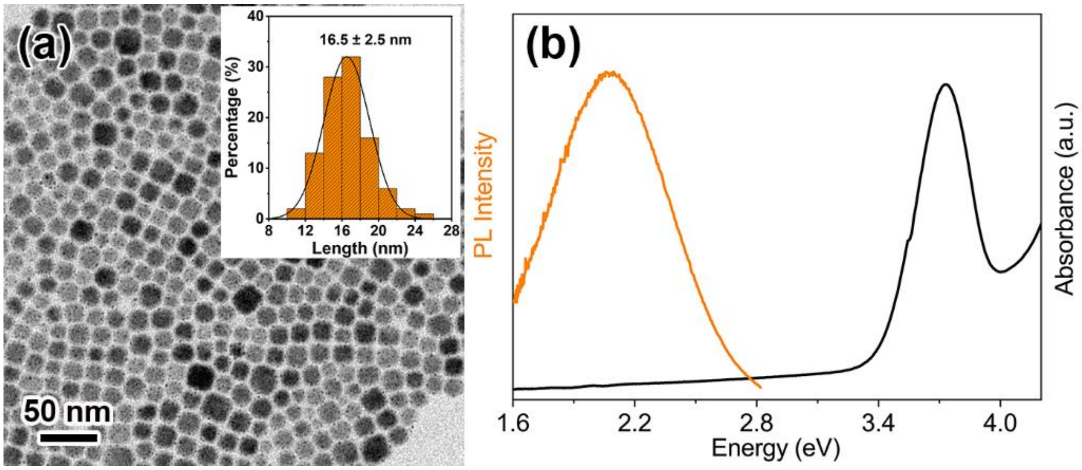

Figure S3. TEM image (a), absorption and PL spectra (b) of Bi-doped $Cs_2Ag_{0.15}Na_{0.85}InCl_6$ NCs synthesized via the procedure reported in our previous work.[10] No Ag^0 LSPR absorption peak is observed at ~2.9 eV (that is 425nm). The PLQY measured for this sample 34±3%.

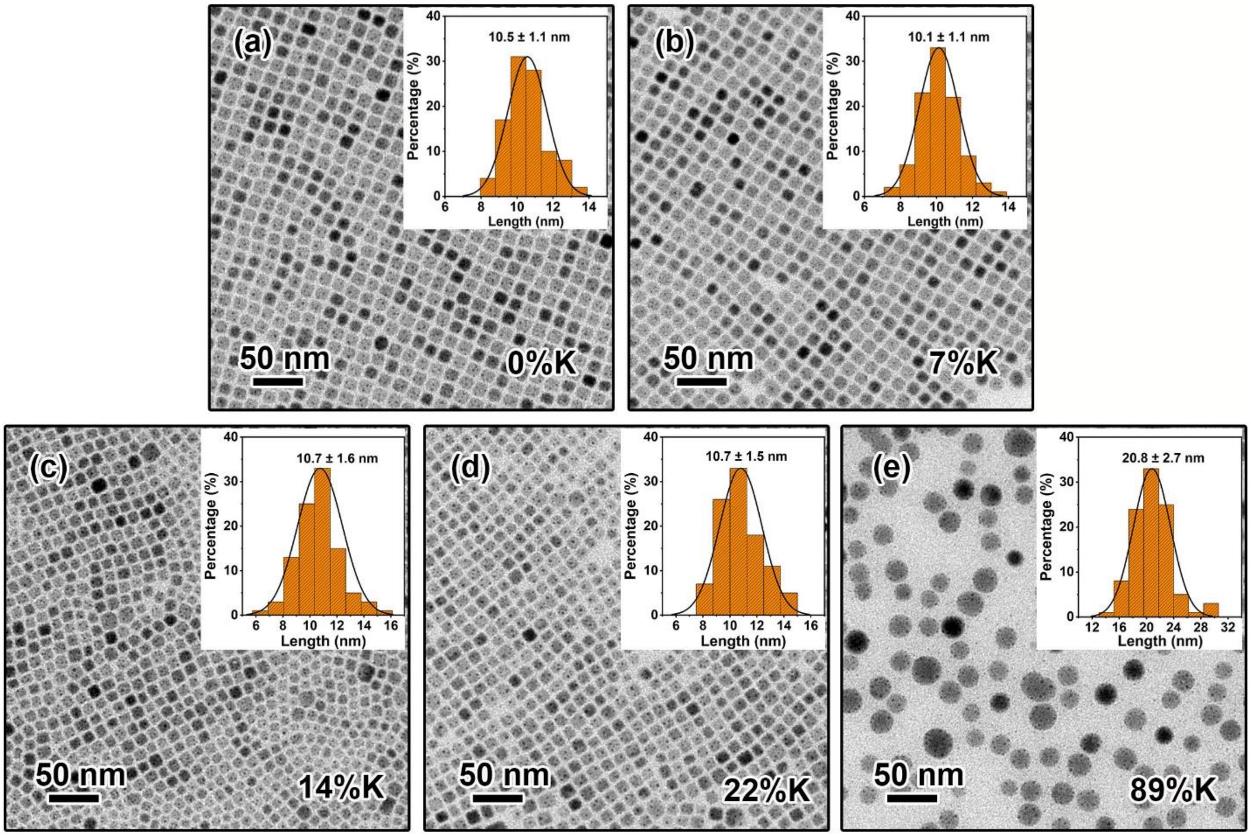

Figure S4. TEM images and corresponding size distribution histograms of Bi-doped $Cs_2Ag_{1-x-y}Na_xK_yInCl_6$ NCs.

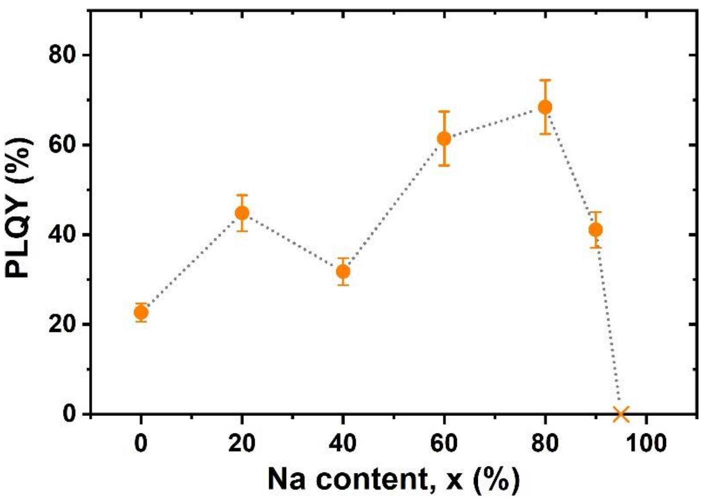

Figure S5. PLQY values measured for Bi-doped $Cs_2Ag_{1-x-y}Na_xK_yInCl_6$ NCs synthesized by fixing the K content (y=0.07) and by varying the Ag/Na precursors ratio.

Table S1. PL lifetime extracted from time-resolved PL decay traces of Bi-doped $Cs_2Ag_{1-x-y}Na_xK_yInCl_6$ NCs shown in Figure 2f.

Sample	PL lifetime (μs)
0%K	2.4
7%K	3.5
14%K	4.1
22%K	4.1
89%K	4.2

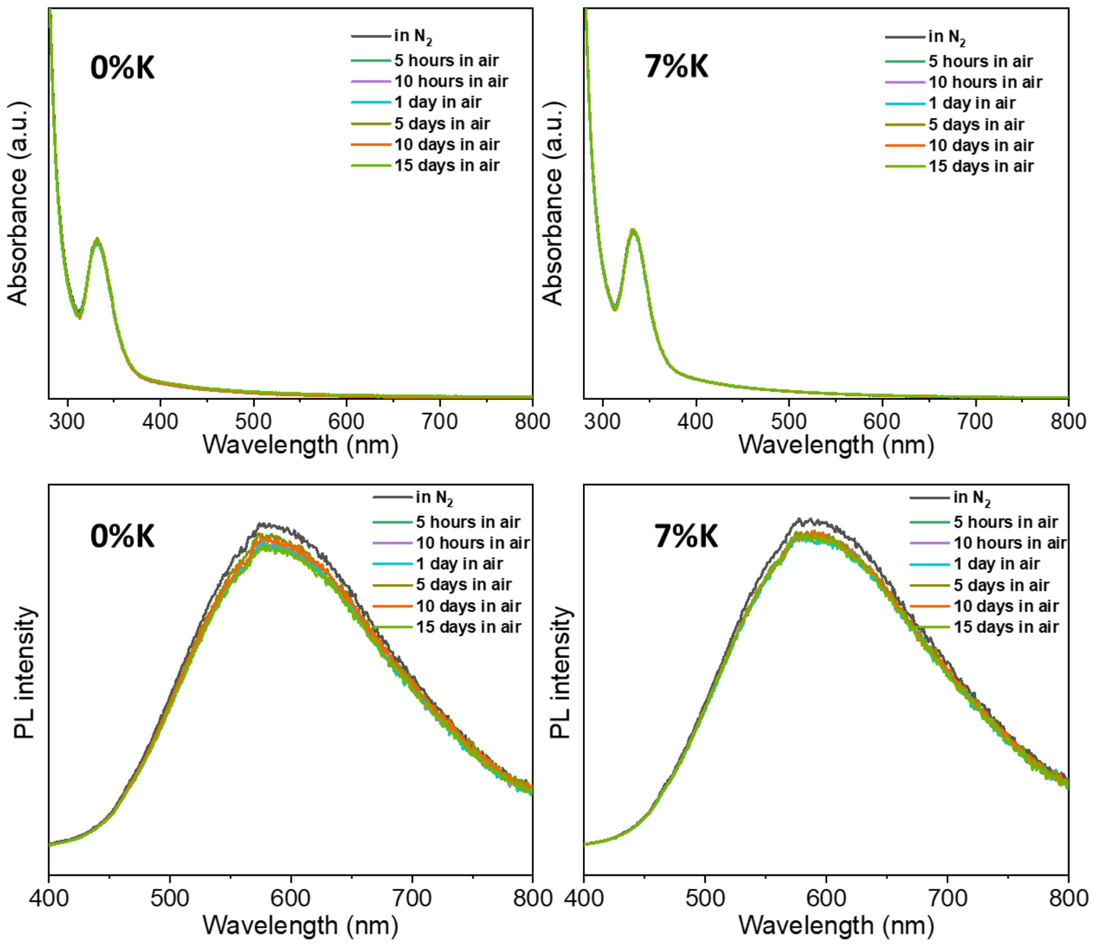

Figure S6. Absorption and PL spectra of 0%K and 7%K NC samples dispersed in toluene and exposed to air up to 15 days.

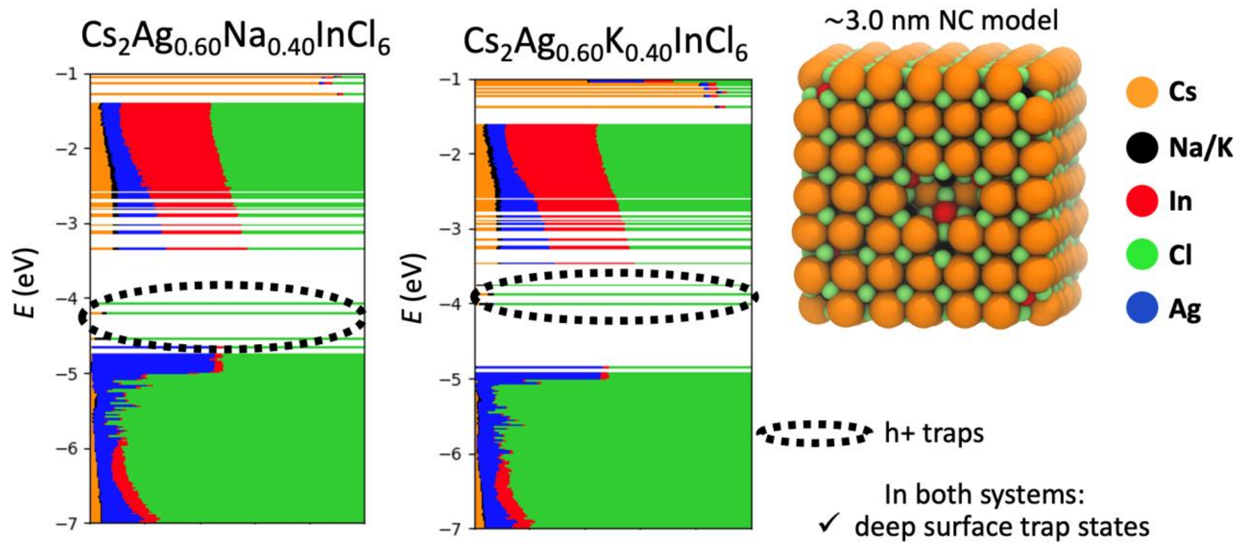

Figure S7. Electronic structure of pure Na and K-alloyed $Cs_2Ag_xNa(K)_{1-x}InCl_6$ double perovskite systems of 3.0 nm in size computed at the DFT/PBE level of theory. Each molecular orbital has been decomposed according to each atom type. In both systems, the removal of CsCl ionic pair from the surface indicate the emergence of deep hole trap states.

Quantitative NMR analysis

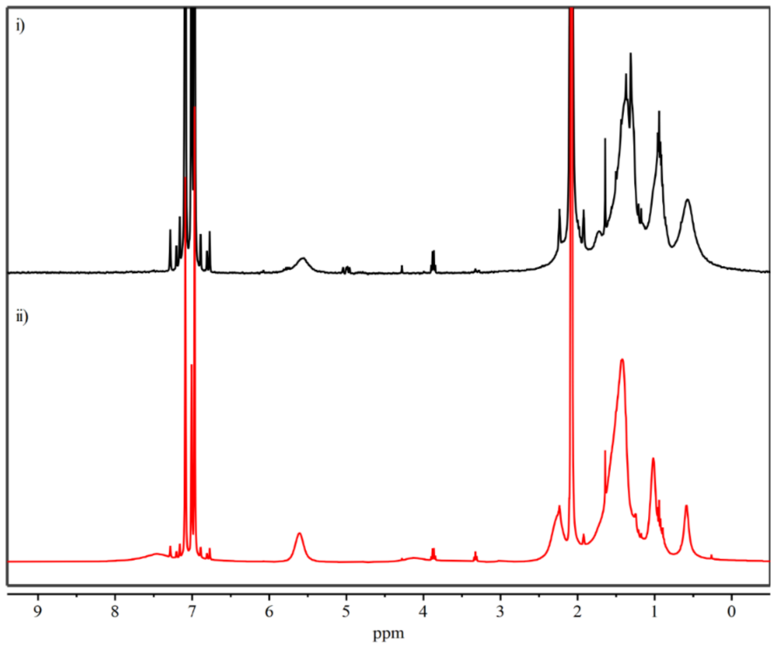

Figure S8. ^1H NMR stacked spectra in toluene-d_8 of i) 0%K NCs in black and ii) 7%K NCs in red. In the ^1H NMR spectra, peak broadening is due to species (namely oleylamine and oleic acid) which are dynamically interacting with the NCs surface.

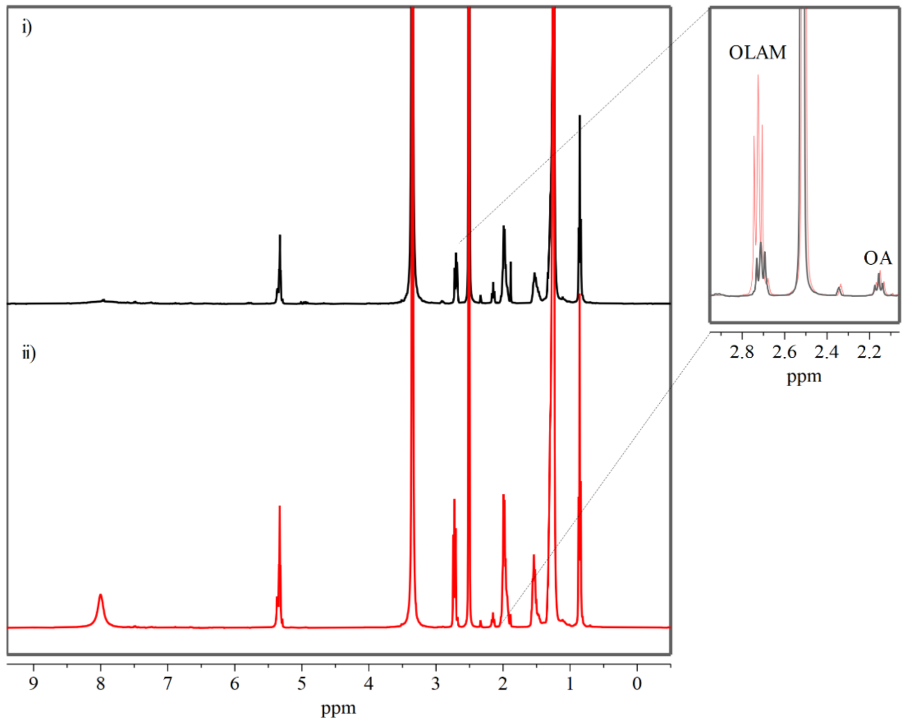

Figure S9. ^{1}H NMR stacked spectra of i) 0%K sample in black and ii) 7%K sample in red, after their dissolution in DMSO-d_6. The inset reports the superimposed ^{1}H spectral region of diagnostic signals of oleylamine (OLAM) and oleic acid (OA).

2D experiments (HSQC and HMBC) were performed to unambiguously ascertain the diagnostic signal of oleylamine (OLAM) and that of oleic acid (OA).

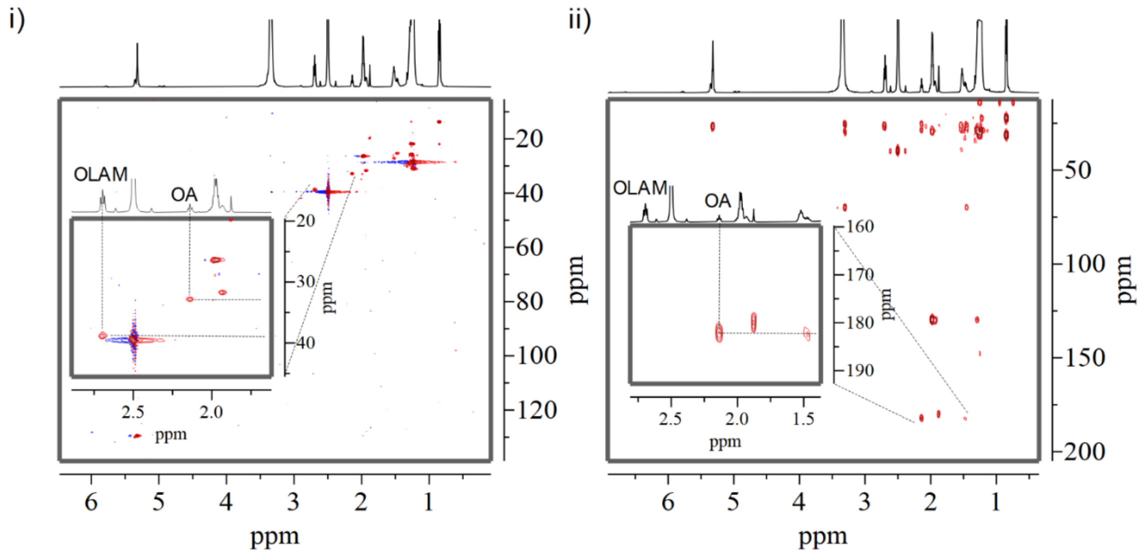

Figure S10. 2D NMR spectra recorded for the 0%K NC sample after dissolution in DMSO-d_6. i) The ^{1}H-^{13}C HSQC (multiplicity edited Heteronuclear Single Quantum Coherence) experiment in which the CH_2 in position α to a nitrogen group (of OLAM) returns a diagnostic cross correlation between the resonances at 2.69 (^{1}H) and 39.0 (^{13}C) ppm. ii) ^{1}H-^{13}C HMBC (Heteronuclear Multiple Bond Correlation) experiment was used to assign the CH_2 in position α to the -COOH group (of OA) by a 1,2-HMBC cross correlation between the resonances of ^{1}H at 2.15 ppm and that of ^{13}C at 181.8 ppm, the latter characteristic of acidic function.

The quantitative NMR analysis of the 0%K NC sample yielded 658 oleylamine and 201 carboxylic acid molecules per NC. Considering that the size of these NCs is 10.52nm (see Figure S4a), corresponding to a surface of 665nm^2, the surface ligands density is 1.3 ligands/nm^2 with 76.6% OLAM and 23.4% OA.

The quantitative NMR analysis of the 7%K NC sample yielded 1283 oleylamine and 156 carboxylic acid molecules per NC. Considering that the size of these NCs is 10.14nm (see Figure S4b), corresponding to a surface of 617nm^2, the surface ligands density is 2.3 ligands/nm^2 with 89.1% OLAM and 10.9% OA.

Surface Sites Occupancy

We start from an ideal atomistic model of a cubic $Cs_2AgNa(K)InCl_6$ NC of 10.5 nm in size, terminated with CsCl species as this is the expect termination of metal halide perovskites systems.[10] The number of atoms for each atom type is listed in Table S2, where we also decompose the number of Cs and Cl at the surface and at the core. A strong assumption for these calculations is that the number of core atoms remains unchanged and fluctuations in their values from the experiments only affects surface atoms (through ion-pair detachments).

We take the number of In atoms as a reference to calculate the number of Cs and Cl atoms, because In is in the core. From the XPS analysis we can estimate the absolute number of Cs and Cl from the measurement of the Cs/In and Cl/In ratios. This allows us, in the real model (see Table S2), to estimate the actual number of surface Cs and Cl ions in the real system, by subtracting the ideal number of Cs and Cl found in the core. From NMR experiments we can also estimate the number of OLAM and OA ligands per NC that are known to replace at the surface Cs and Cl ion sites, respectively. Because of this, we can estimate, in the real system, the total number of surface A (Cs + OLAM) and X (Cl + OA) sites occupied. Considering that we know in the ideal system how many of surface A and X ions should be occupied in the ideal system, we can provide an estimate for the A and X site real occupancy.

Table S2. List of parameters employed for the determination of Surface Sites Occupancy

	$Cs_2(Ag,Na)InCl_6$	$Cs_2(Ag,Na,K)InCl_6$
**Ideal Model**		
n^0 In	4000	4000
n^0 core Cs	6859	6859
n^0 core Cl	22800	22800
n^0 surface A	2402	2402
n^0 surface X	2400	2400
**XPS**		
Cs/In	1.76	1.92
Cl/In	5.78	6
n^0 total Cs	7040	7680
n^0 total Cl	23120	24000
**NMR**		
n^0 OLAM	658	1283
n^0 OA	201	156
**Real Model**		
n^0 In	4000	4000
n^0 core Cs	6859	6859
n^0 core Cl	22800	22800
n^0 surface Cs = (n^0 total Cs - n^0 core Cs)	181	821
n^0 surface Cl = (n^0 total Cl - n^0 core Cl)	320	1200
n^0 surface OLAM	658	1283
n^0 surface OA	201	156
n^0 surface A = n^0 surface Cs + n^0 surface OLAM	839	2104
n^0 surface X = n^0 surface Cl + n^0 surface OA	521	1356
**Surface Occupancy**		
% A site = n^0 surface A / n^0 ideal surface A	35%	88%
% X site = n^0 surface X / n^0 ideal surface X	22%	57%

References

(1) Fairley, N.; Fernandez, V.; Richard-Plouet, M.; Guillot-Deudon, C.; Walton, J.; Smith, E.; Flahaut, D.; Greiner, M.; Biesinger, M.; Tougaard, S.; Morgan, D.; Baltrusaitis, J., Systematic and Collaborative Approach to Problem Solving Using X-Ray Photoelectron Spectroscopy. *Applied Surface Science Advances* **2021,** *5*, 100112.

(2) Kresse, G.; Hafner, J., Ab Initio Molecular Dynamics for Liquid Metals. *Phys. Rev. B* **1993,** *47*, 558-561.

(3) Kresse, G.; Furthmüller, J., Efficiency of Ab-Initio Total Energy Calculations for Metals and Semiconductors Using a Plane-Wave Basis Set. *Computational Materials Science* **1996,** *6*, 15-50.

(4) Kresse, G.; Joubert, D., From Ultrasoft Pseudopotentials to the Projector Augmented-Wave Method. *Phys. Rev. B* **1999,** *59*, 1758-1775.

(5) Perdew, J. P.; Burke, K.; Ernzerhof, M., Generalized Gradient Approximation Made Simple [Phys. Rev. Lett. 77, 3865 (1996)]. *Phys. Rev. Lett.* **1997,** *78*, 1396-1396.

(6) VandeVondele, J.; Hutter, J., Gaussian Basis Sets for Accurate Calculations on Molecular Systems in Gas and Condensed Phases. *J. Chem. Phys.* **2007,** *127*, 114105.

(7) Hutter, J.; Iannuzzi, M.; Schiffmann, F.; VandeVondele, J., Cp2k: Atomistic Simulations of Condensed Matter Systems. *WIREs Computational Molecular Science* **2014,** *4*, 15-25.

(8) Wu, P. S. C.; Otting, G., Rapid Pulse Length Determination in High-Resolution NMR. *J. Magn. Reson.* **2005,** *176*, 115-119.

(9) Wider, G.; Dreier, L., Measuring Protein Concentrations by Nmr Spectroscopy. *J. Am. Chem. Soc.* **2006,** *128*, 2571-2576.

(10) Zhang, B.; Wang, M.; Ghini, M.; Melcherts, A. E. M.; Zito, J.; Goldoni, L.; Infante, I.; Guizzardi, M.; Scotognella, F.; Kriegel, I.; De Trizio, L.; Manna, L., Colloidal Bi-Doped $Cs_2Ag_{1-x}Na_xInCl_6$ Nanocrystals: Undercoordinated Surface Cl Ions Limit Their Light Emission Efficiency. *ACS Mater. Lett.* **2020,** *2*, 1442-1449.

(11) Kaushik, V. K., XPS Core Level Spectra and Auger Parameters for Some Silver Compounds. *Journal of Electron Spectroscopy and Related Phenomena* **1991,** *56*, 273-277.

(12) Ferraria, A. M.; Carapeto, A. P.; Botelho do Rego, A. M., X-Ray Photoelectron Spectroscopy: Silver Salts Revisited. *Vacuum* **2012,** *86*, 1988-1991.
